# Validating the risk of hypoparathyroidism after total thyroidectomy in a population-based cohort: plea for improved follow-up

**DOI:** 10.1093/bjs/znad366

**Published:** 2023-11-23

**Authors:** Matilda Annebäck, Carolina Osterman, Jesper Arlebrink, Simon Mellerstedt, Nicolas Papathanasakis, Göran Wallin, Ola Hessman, Maria Annerbo, Olov Norlén

**Affiliations:** Department of Surgical Sciences, Uppsala University, Uppsala, Sweden; Department of Surgery, Gävle Hospital, Gävle, Sweden; Department of Surgery, Karlstad Central Hospital, Karlstad, Sweden; Department of Surgery, Falu Hospital, Karlstad, Sweden; Department of Surgery, Örebro University Hospital, Örebro, Sweden; Department of Surgery, Örebro University Hospital, Örebro, Sweden; Department of Surgery, Västerås Central Hospital, Västerås, Sweden; Department of Surgery, Falu Hospital, Karlstad, Sweden; Department of Surgical Sciences, Uppsala University, Uppsala, Sweden

## Abstract

**Background:**

A previous nationwide study from Sweden showed that the rate of permanent hypoparathyroidism is high and under-rated in the Swedish Quality Register. This retrospective population-based study aimed to validate the rate and diagnosis of permanent hypoparathyroidism found in the previous study. A secondary aim was to assess the relationship between the rate of low parathyroid hormone (PTH) levels within 24 h after surgery and the rate of permanent hypoparathyroidism.

**Methods:**

All patients who underwent total thyroidectomy from 2005 to 2015 in a region of Sweden were included. Data were retrieved from local health records, the National Patient Registry, the Swedish Prescribed Drug Registry, and the Swedish Quality Register. A strict definition of permanent hypoparathyroidism was used, including biochemical data and attempts to stop the treatment.

**Results:**

A total of 1636 patients were included. Altogether, 143 patients (8.7 per cent) developed permanent hypoparathyroidism. Of these, 102 (6.2 per cent) had definitive permanent hypoparathyroidism, whereas 41 (2.5 per cent) had possible permanent hypoparathyroidism, because attempts to stop the treatment were lacking (28) or patients were lost to follow-up (13). The agreement between the Swedish Quality Register and the chart review was 29.3 per cent. A proportion of 23.2 per cent with a PTH level below the reference value corresponded to a 6.7 per cent rate of permanent hypoparathyroidism.

**Conclusion:**

The risk of permanent hypoparathyroidism after total thyroidectomy is high. Some patients are overtreated because attempts to stop the treatment are lacking. Quality registers might underestimate the risk of permanent hypoparathyroidism. Approximately one-quarter of all patients with low PTH levels immediately after surgery developed permanent hypoparathyroidism.

## Teaser

This retrospective population-based cohort study from Sweden validated the rate and diagnosis of permanent hypoparathyroidism, using a strict definition including both biochemical data and attempts to stop the treatment. The rate of permanent hypoparathyroidism is high, and some patients might be misdiagnosed and overtreated because attempts have not been made to stop the treatment. One-quarter of all patients with an early low PTH level below the reference range develop permanent hypoparathyroidism.

## Introduction

Hypoparathyroidism is the most frequent complication after total thyroidectomy^1^. The complication may be transient or permanent. The rate of permanent hypoparathyroidism varies widely between studies, ranging from 1 to 12.5 per cent^2,3^. The type and extent of surgery, operative technique, surgeon experience and operative volume, and patient-related factors influence the risk of permanent hypoparathyroidism in patients undergoing thyroidectomy^4–8^. The wide range can also be explained by the strikingly varying definitions of permanent hypoparathyroidism^9–11^.

Permanent hypoparathyroidism can be defined either based on biochemical findings, or by the need for treatment with supplements to maintain normocalcaemia. A common definition is the need for calcium and/or active vitamin D at 6 or 12 months after surgery. Some patients recover from transient hypoparathyroidism more than 6 months after surgery, suggesting use of the 12-month deadline.

Although conventional therapy effectively raises serum calcium levels, high doses of calcium can lead to gastrointestinal side-effects and hypercalciuria, with an increased risk of kidney stones and renal insufficiency^12,13^. Other long-term consequences of permanent hypoparathyroidism are calcification of basal ganglia, increased risk of infections, psychiatric disorders, as well as increased risk of malignancy and death^14–16^. Patients who develop permanent hypoparathyroidism should receive appropriate follow-up to monitor for long-term complications related therapy with supplements. Furthermore, the diagnosis of permanent hypoparathyroidism has an economic impact, with patients needing lifelong treatment and regular follow-up. To avoid overtreatment and incorrect diagnosis of permanent hypoparathyroidism, it is crucial that attempts are made to stop the treatment.

Multicentre and national register studies have consistently reported higher rates of permanent hypoparathyroidism than single-centre studies^10^. In national quality registers, long-term follow-up is often incomplete, with a large amount of missing data, making information on long-term complications less reliable. This has been illustrated by the UK Registry of Endocrine and Thyroid Surgeons, in which almost 22 per cent of patients had missing data on treatment with supplements at 6-month follow-up after thyroidectomy^17^. A previously published Swedish nationwide study^2^, including 7852 patients, showed a rate of permanent hypoparathyroidism of 12.5 per cent in patients undergoing total thyroidectomy for benign thyroid disease. In this study, permanent hypoparathyroidism was defined by prescription and dispensing of calcium and/or active vitamin D in the Swedish Prescribed Drug Register (SPDR) more than 12 months after surgery. The study showed a discrepancy in the rate of permanent hypoparathyroidism compared with data reported in the Swedish Quality Register for Thyroid, Parathyroid and Adrenal Surgery (SQRTPA). Moreover, the SQRTPA only reported 178 (18.1 per cent) of the 983 patients with permanent hypoparathyroidism according to the set definition in the study as permanently hypoparathyroid.

To exclude permanent hypoparathyroidism adequately, both 100 per cent complete long-term follow-up and a test of stopping all calcium and active vitamin D treatment is required, but to achieve this is challenging in most centres. As such, the inherent difficulty in accurately establishing the proportion of patients who develop permanent hypoparathyroidism makes it problematic to study any attempts to decrease this fairly unusual complication. An immediate postoperative surrogate marker that could predict the true rate of permanent hypoparathyroidism would be very valuable for both studies and for quality monitoring purposes.

The aim of this study was to investigate the rate of permanent hypoparathyroidism, defined as the need for calcium and/or active vitamin D more than 12 months after surgery, to evaluate the follow-up and diagnosis of permanent hypoparathyroidism in a population-based setting, and to validate previously published data^2^. The hypothesis was that patients may be misdiagnosed with permanent hypoparathyroidism and thus overtreated, because of lack of attempts to unwind the treatment. The secondary aims were to assess the relationship between an immediate postoperative surrogate marker, serum parathyroid hormone (PTH), and the risk of permanent hypoparathyroidism in the total cohort and to validate the reported data on complications in the SQRTPA.

## Methods

### Ethical approval

Ethical approval for the study was obtained from the Regional Ethical Review Board of Uppsala on 30 November 2016 (diary no. 2016/479) and from the Swedish Ethical Review Authority on 23 November 2020 (diary no. 2020-05744).

### Patients

This population-based retrospective cohort study was based on a population-based nationwide cohort study of the rate of permanent hypoparathyroidism after total thyroidectomy for benign thyroid disease^2^. The nationwide study included all patients who underwent total thyroidectomy in Sweden between 2005 and 2015. The patients were identified through the SQRTPA and/or the Swedish National Patient Register (NPR). Data on prescription and dispensing of calcium and active vitamin D were extracted from the SPDR.

**Fig. 1:**
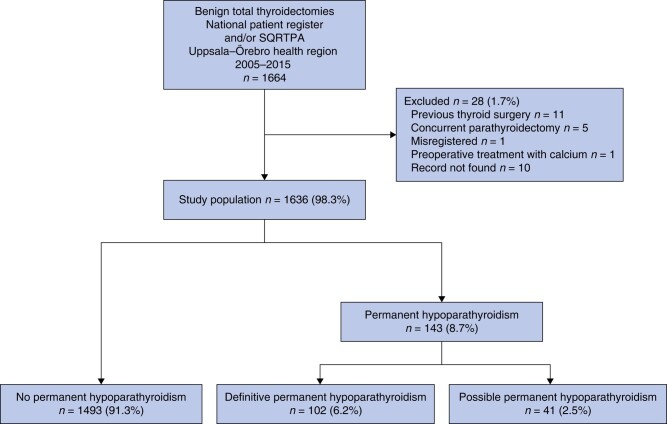
Study flow chart SQRTPA, Swedish Quality Register for Thyroid, Parathyroid and Adrenal Surgery.

From the present subcohort, all patients who underwent surgery at two University hospitals (Uppsala and Örebro) and the four surrounding regional hospitals (Falun, Gävle, Karlstad, and Västerås) were included in the present study (*Fig. S1*). The population base of the study encompassed roughly 1.8 million (18 per cent) of the entire Swedish population (2015 data). Local hospital records were reviewed at the participating hospitals. Exclusion criteria were: preoperative treatment with calcium and/or vitamin D, thyroid cancer, previous thyroid surgery, previous or concurrent parathyroid surgery, previous or concurrent lymph node dissection, and sternotomy. Patients were also excluded if the operation date differed by more than 30 days in the SQRTPA and NPR, as were those who had other types of thyroid surgery registered in the SQRTPA, and those with missing health records at the local hospitals (*Fig. 1*).

A structured form was used for the collection of data from patients’ electronic health records: sex; age; indication for surgery; number of parathyroid glands identified; perioperative and postoperative complications, including need for reoperation and readmission within 30 days; preoperative and postoperative calcium and PTH values (postoperative day 1, day 2–3, week 1–6, 6 months, 6–12 months, and 12–24 months); treatment with calcium and active vitamin D; indication for the treatment with calcium and active vitamin D; and attempts to stop the treatment.

### Definition of primary outcome

Permanent hypoparathyroidism was divided into two categories: definitive permanent hypoparathyroidism and possible permanent hypoparathyroidism (*Fig. 1*).

### Definitive permanent hypoparathyroidism

Patients were considered to have definitive permanent hypoparathyroidism if they met one or more of the following criteria between 12 and 24 months after surgery: low intact PTH level (below the lower limit of the local hospital’s standard) accompanied by hypocalcaemia (albumin-corrected or ionized calcium level below the lower limit of the local hospital’s standard); hypocalcaemia without an appropriate physiological increase in PTH level; and/or at least one unsuccessful attempt at stopping treatment with calcium and/or active vitamin D supplements.

### Possible permanent hypoparathyroidism

Patients were considered to have possible permanent hypoparathyroidism if they remained on calcium and/or active vitamin D supplementation but did not fulfil any of the three criteria above between 12 and 24 months after surgery, or if they remained on supplements but were lost to follow-up before 12 months after surgery.

### No permanent hypoparathyroidism

Patients who did not fulfil any of the above criteria and were not treated with calcium and/or active vitamin D supplements during the interval between 12 and 24 months after surgery were considered to not have permanent hypoparathyroidism.

### Calculation of secondary outcomes

Comparisons between patients with and those without permanent hypoparathyroidism were made to analyse patient characteristics, and the relationship between immediate low postoperative PTH level (defined as serum PTH level 1–24 h after surgery) and definitive permanent hypoparathyroidism. Patients with possible permanent hypoparathyroidism were excluded from this analysis because the diagnosis was uncertain.

### Definition of other complications

Data on postoperative bleeding and surgical-site infection (SSI) were retrieved from the local hospital records. Postoperative bleeding was defined by the need for reoperation for bleeding. The diagnosis of SSI was left to the discretion of the attending surgeon or physician. Patients were considered to have an SSI if this was noted in the charts within 30 days of surgery. Permanent recurrent laryngeal nerve (RLN) injury was defined by vocal cord paresis or palsy on laryngoscopy more than 12 months after operation. Patients without a documented laryngoscopy in the local health records were registered as having missing data.

### Statistical analysis

Continuous data are presented as mean(s.d.). Differences between groups were assessed by means of the χ^2^ or Student’s *t* test. Two-sided *P* < 0.050 was considered statistically significant. SPSS^®^ software (IBM, Armonk, NY, USA) was used for statistical analysis.

## Results

Of 7852 patients included in the nationwide cohort, 1664 underwent total thyroidectomy at the participating hospitals. After excluding those who had undergone thyroid surgery previously (11 patients), or previous or concurrent parathyroid surgery (5), been misregistered as having total thyroidectomy (1), received preoperative treatment with calcium (1), or medical records missing (10), a total of 1636 patients were eligible for analysis (*Fig. 1*).

The mean(s.d.) age at surgery was 45(16) years and 83.7 per cent of the patients were women. In total, 1084 patients (66.3 per cent) had surgery owing to thyrotoxicosis and 493 (30.1 per cent) because of compression symptoms. Parathyroid autotransplantation was performed in 289 (17.7 per cent) (*Table 1*).

**Table 1:** Baseline characteristics of study cohort

	No. of patients* (*n* = 1636)
**Age (years), mean(s.d.)**	45(16)
**Age (years)**	
< 40	655 (40.0)
40–60	672 (41.1)
> 60	309 (18.9)
**Sex ratio (M:F)**	266 : 1370
**Indication for surgery**	
Thyrotoxicosis	1084 (66.3)
Compression symptoms	493 (30.1)
Excluding malignancy	41 (2.5)
Other reasons	18 (1.1)
**No. of parathyroid glands identified during surgery**	
0	18 (1.1)
1	154 (9.4)
2	569 (34.8)
3	402 (24.6)
≥ 4	481 (29.4)
Missing data	12 (0.7)
**Parathyroid autotransplantation**	
Yes	289 (17.7)
No	1347 (82.3)
**Reoperation for bleeding**	
Yes	27 (1.7)
No	1609 (98.3)
**Surgical-site infection**	
Yes	25 (1.5)
No	1611 (98.5)
**Permanent recurrent laryngeal nerve injury**	
Yes	9 (0.6)
No	761 (46.5)
Missing data	866 (52.9)
**Readmission within 30 days after surgery**	
Yes	31 (1.9)
No	1605 (98.1)
**Reason for readmission**	
Rebleeding	7 (0.4)
Infection	6 (0.4)
Hypocalcaemia	18 (1.1)
No readmission	1605 (98.1)

^*^Values are *n* (%) unless otherwise indicated.

### Primary outcome

A total of 143 of 1636 patients (8.7 per cent) developed definitive or possible permanent hypoparathyroidism according to the study definitions. Of these, 102 patients (6.2 per cent) had definitive permanent hypoparathyroidism, whereas 41 (2.5 per cent) had possible permanent hypoparathyroidism, because attempts to stop the treatment were lacking (28) or they were lost to follow-up (13) (*Fig. 1*).

### Treatment of patients with definitive permanent hypoparathyroidism

In the group with definitive permanent hypoparathyroidism, 47 of 102 patients (46.1 per cent) were treated with both calcium and active vitamin D, whereas 24 (23.5 per cent) had active vitamin D monotherapy, and 23 (22.5 per cent) were treated with calcium supplements alone. Supplements were later reinstated in an additional three patients (2.9 per cent) who had hypocalcaemia and low PTH levels more than 24 months after surgery, although they had no treatment 12–24 months after surgery. Five patients (4.9 per cent) had biochemical evidence of permanent hypoparathyroidism, although data on medical treatment were missing from the patient records.

### Treatment of patients with possible permanent hypoparathyroidism

A slightly different pattern was found in the group with possible permanent hypoparathyroidism, in which 13 patients (32 per cent) were treated with calcium only, 8 (20 per cent) with active vitamin D only, and 7 (17 per cent) with a combination of these supplements. As mentioned above, 13 patients (32 per cent) had treatment for hypoparathyroidism after surgery but were lost to follow-up, and so information was missing regarding treatment 12–24 months after surgery.

### Secondary outcomes

#### Baseline differences between patients with and without permanent hypoparathyroidism

Comparison of patients with *versus* without definitive permanent hypoparathyroidism showed was no differences in patient characteristics, indication for surgery, number of parathyroid glands identified, parathyroid autotransplantation, reoperation for bleeding, or SSI (*Table 2*). Patients who developed definitive permanent hypoparathyroidism were more likely to have been readmitted owing to symptomatic hypocalcaemia than those who did not develop permanent hypoparathyroidism (*P* < 0.001) (*Table 2*).

**Table 2:** Clinical characteristics of patients with and without definitive permanent hypoparathyroidism

	Definitive permanent hypoparathyroidism (*n* = 102)	No permanent hypoparathyroidism (*n* = 1493)	P*
**Age (years)**			0.055
< 40	50 (49.0)	590 (39.5)	
40–60	41 (40.2)	616 (41.3)	
> 60	11 (10.8)	287 (19.2)	
**Sex ratio (M:F)**	19 : 83	242 : 1251	0.523
**Indication for surgery**			0.502
Thyrotoxicosis	67 (65.7)	993 (66.5)	
Compression symptoms	32 (31.4)	447 (29.9)	
Excluding malignancy	1 (1.0)	40 (2.7)	
Other reasons	2 (1.9)	13 (0.9)	
**No. of parathyroid glands identified**			0.430
0	1 (1.0)	17 (1.1)	
1	16 (15.7)	136 (9.1)	
2	36 (35.3)	520 (34.8)	
3	24 (23.5)	364 (24.4)	
≥ 4	24 (23.5)	446 (29.9)	
Missing data	0 (0)	2 (0.1)	
**Parathyroid autotransplantation**			0.367
Yes	21 (20.6)	257 (17.2)	
No	80 (78.4)	1231 (82.5)	
**Reoperation for bleeding**			1.000
Yes	1 (3.7)	26 (96.3)	
No	101 (6.4)	1467 (93.6)	
**Surgical-site infection**			1.000
Yes	1 (1.0)	21 (1.4)	
No	101 (99.0)	1472 (98.6)	
**Permanent recurrent laryngeal nerve injury**			0.236
Yes	0 (0)	9 (0.6)	
No	54 (52.9)	672 (45.0)	
Missing data	48 (47.1)	812 (54.4)	
**Readmission within 30 days after surgery**			< 0.001
Yes	7 (6.9)	22 (1.5)	
No	95 (93.1)	1471 (98.5)	
**Readmission owing to hypocalcaemia**			< 0.001
Yes	6 (5.9)	11 (0.7)	
No	96 (94.1)	1482 (99.3)	

Values are *n* (%). Forty-one patients with possible permanent hypoparathyroidism are not included in the table. *χ^2^ test.

#### Immediate postoperative parathyroid hormone as a surrogate marker for permanent hypoparathyroidism

An immediate postoperative PTH measurement was not clinical routine in most of the participating centres during the first years of the study, and the availability differed among the participating hospitals. A total of 624 patients (39.1 per cent) had a documented PTH value within 1–24 h after surgery. The rate of definitive permanent hypoparathyroidism among the 624 patients was 6.7 per cent (42 patients). Of these 624 patients, 145 (23.2 per cent) had a PTH level below the lower limit of the current local reference range (*Table 3*). In other words, 23.2 (95 per cent c.i. 20.0 to 26.8) per cent of patients with a low PTH level corresponded to a rate of definitive permanent hypoparathyroidism of 6.7 (4.9 to 9.0) per cent. The remaining 3 of the 42 patients with definitive permanent hypoparathyroidism had an immediate PTH level that was within the normal reference range, albeit just above the lower limit (PTH less than 5 per cent above lower reference limit).

**Table 3:** Parathyroid hormone levels 1–24 h after surgery

PTH levels 1–24 h after surgery	Definitive permanent hypoparathyroidism (*n* = 42)*	No permanent hypoparathyroidism (*n* = 582)†	*P*‡
**Below lower reference limit**	39 (26.9)	106 (73.1)	< 0.001
**Within or above reference range**	3 (0.6)	476 (99.4)	

Values are *n* (%). Data on parathyroid hormone (PTH) missing for *60 and †911 patients.

#### Agreement between present cohort and national registers regarding permanent hypoparathyroidism

**Fig. 2:**
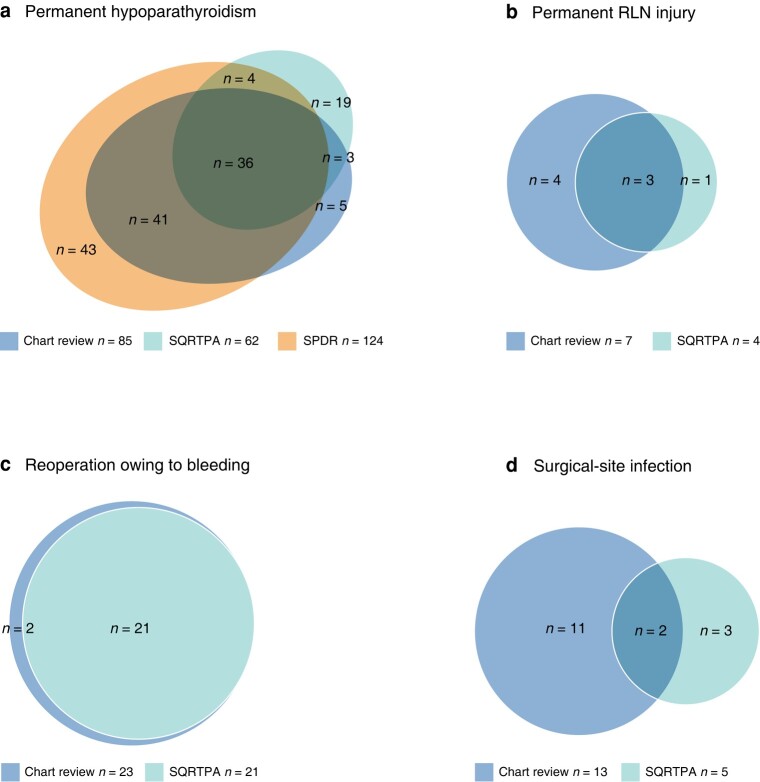
Agreement on complications between chart review and national registers **a** Permanent hypoparathyroidism, **b** permanent recurrent laryngeal nerve (RLN) injury, **c** reoperation owing to bleeding, and **d** surgical-site infection. SQRTPA, Swedish Quality Register for Thyroid, Parathyroid and Adrenal Surgery; SPDR, Swedish Prescribed Drug Register.

Some 1306 patients (79.8 per cent) were registered in the SQRTPA during the study interval. Of these, 85 (6.5 per cent) were found to have definitive permanent hypoparathyroidism according to the study definition, compared with 62 patients (4.7 per cent) registered with permanent hypoparathyroidism in the SQRTPA. Moreover, 124 patients (9.5 per cent) had treatment with calcium/and or active vitamin D according to the SPDR (*Fig. 2*). The agreement between definitive hypoparathyroidism according to the study definition and the data reported in the SQRTPA was 29.3 per cent.

#### Other surgical complications

After operation, 27 patients (1.7 per cent) underwent surgery because of bleeding and 25 (1.5 per cent) developed SSI (*Table 1*). Some 31 patients (1.9 per cent) were readmitted within 30 days after surgery, with hypocalcaemia being the most common reason for readmission (*Table 1*). Permanent RLN injury was found in nine patients (0.5 per cent) in the study cohort, although approximately half of the included patients had data missing on postoperative laryngoscopy (*Table 1*). The agreement between the data collected in the chart review and those reported in the SQRTPA was 91.3 per cent for reoperation owing to rebleeding, 14.3 per cent for SSI, and 60.0 per cent for permanent RLN injury (*Fig. 2*).

## Discussion

In this population-based study of 1636 patients who had total thyroidectomy for benign thyroid disease, 8.7 per cent developed definitive or possible permanent hypoparathyroidism according to the study definition. This demonstrates that the definition of permanent hypoparathyroidism used in the previously published national cohort study of the same patients led to an adequate approximation, even though the rate of permanent hypoparathyroidism was slightly higher when the definition from the register-based study was used^2^.

A rate of 8.7 per cent (143 patients) with permanent hypoparathyroidism is quite high, but in keeping with several other large, national studies, which reported rates ranging from 9 to 12.1 per cent^4,17,18^. Scrutinizing further, 6.2 per cent (102 patients) were found to have definitive permanent hypoparathyroidism, and an additional 2.5 per cent (41) to have possible permanent hypoparathyroidism. Of these 41 patients, 28 remained on treatment with calcium and/or active vitamin D 12–24 months after surgery, but had no documented attempts to stop the treatment. Therefore, these patients may or may not have had permanent hypoparathyroidism. This is indeed problematic because such patients be overtreated with supplements, which generates costs for both the patient and the healthcare system, as well as possible treatment side-effects and long-term consequences. The results of this study highlight the importance of a strict diagnosis of permanent hypoparathyroidism, using both biochemical findings and attempts to stop the treatment, to avoid overtreatment and misdiagnosis.

In this study, it was proposed that the rate of patients with a low postoperative PTH level within 1–24 h after surgery may correspond to the rate of permanent hypoparathyroidism in a cohort. The results showed that 23.2 (95 per cent c.i. 20.0 to 26.8) per cent of patients with a low immediate PTH value corresponded to 6.7 (4.9 to 9.0) per cent developing definitive permanent hypoparathyroidism. Additionally, almost all patients who eventually developed permanent hypoparathyroidism had an immediate PTH level below the lower reference limit, or in occasional patients just above it, and as such follow-up may be concentrated on these patients. Moreover, 26.9 (19.9 to 34.9) per cent of all patients with an immediate low PTH level developed definitive permanent hypoparathyroidism. Given that the correlation between the proportion of patients with immediate low postoperative PTH levels and permanent hypoparathyroidism is constant, extrapolating this result would suggest that a rate of 30 per cent of patients with an immediate low postoperative PTH level would correspond to a rate of 8.0 (6.0 to 10.5) per cent developing permanent hypoparathyroidism. Furthermore, 10 per cent with a low PTH value would correspond to a 2.9 (1.9 to 3.5) per cent rate, and 5 per cent to a 1.4 (1.0 to 1.7) per cent rate, of permanent hypoparathyroidism. Of course, the relationship between low immediate PTH levels after surgery and the rate of definitive permanent hypoparathyroidism needs to be validated externally in other cohorts, although a similar association has already been suggested by Riordan *et al*.^19^ and Ritter *et al.*^20^. In the study conducted by Ritter *et al*., 10 per cent of patients with a PTH level below the normal reference range within 24 h after surgery developed permanent hypoparathyroidism, a lower proportion than in the present study. It should, however, be noted Ritter *et al*. defined permanent hypoparathyroidism as a PTH value below 10 pg/ml and the need for calcitriol or more than 2000 mg daily calcium supplementation to avoid symptoms of hypocalcaemia. The more inclusive definition of definitive permanent hypoparathyroidism in the present study may account for the higher proportion of patients diagnosed with permanent hypoparathyroidism.

If the relationship between low immediate PTH level and rate of permanent hypoparathyroidism holds in other studies with equally strict definitions of permanent hypoparathyroidism and rigorous follow-up, the rate of low PTH level after surgery may be used as a surrogate marker for the rate of permanent hypoparathyroidism within a unit or patient category. As such, this would offer a simple way of evaluating new techniques used to lower the risk of permanent hypoparathyroidism in patients undergoing total thyroidectomy. In any case, a very high proportion of patients with an immediate postoperative PTH value within the normal reference range after thyroid surgery would definitely be an indicator of adequate parathyroid sparing, as such patients hardly ever develop postoperative permanent hypoparathyroidism. Surgical volume and technique have been shown previously to affect the risk of hypoparathyroidism, although far from all centres reach the numbers needed for the lowest level of permanent hypoparathyroidism risk^6,8^. Over recent years, new technologies, such as indocyanine green fluorescence angiography and parathyroid autofluorescence, have emerged as technical tools that are used to avoid injuring the parathyroid glands and their blood supply during surgery, although the impact on permanent hypoparathyroidism after surgery is still under debate^21–23^. An easy, immediate method using a surrogate marker to approximate the rate of permanent hypoparathyroidism without the need for long follow-up would greatly facilitate both quality control in centres performing thyroidectomy as well as aid in the scientific assessment of new techniques.

The agreement between the data reported in the SQRTPA and the data collected from medical records was found to be high for reoperation for rebleeding, but it was low for follow-up data recording SSI, permanent RLN injury, and permanent hypoparathyroidism (*Fig. 2*). The results of this study imply that follow-up data on these complications in the SQRTPA should be interpreted with some caution. The study highlights the importance of considering when and how data are collected when assessing surgical outcome. Evaluating long-term follow-up data in quality registers poses several challenges, including managing incomplete or missing data and addressing biases that may arise from self-reported information. The accuracy of long-term follow-up data can also be influenced by the completeness of data entry of different healthcare providers. The diagnosis of permanent hypoparathyroidism can be demanding both owing to the long-term follow-up needed, but also in identification, particularly in patients with relative hypoparathyroidism. Relative hypoparathyroidism is a term used when the levels of PTH are normal but insufficient to keep calcium within normal limits. Healthcare providers may mistakenly assume that calcium regulation is normal and thus neglect the underlying calcium dysregulation. The clinical significance of relative hypoparathyroidism remains uncertain because of a lack of comprehensive research conducted to thoroughly explore this condition.

The disagreement between the rate of permanent hypoparathyroidism found in this study and the data reported in the SQRTPA (*Fig. 2*) can possibly be explained partly by the difference in definitions used. In the present study, permanent hypoparathyroidism was defined as parathyroid dysfunction more than 12 months after surgery, whereas the SQRTPA uses a cut-off of 6 months after surgery to define permanent hypoparathyroidism. The fact that some patients recover their parathyroid function after 6 months could explain the proportion of patients with permanent hypoparathyroidism registered solely in the SQRTPA. Additionally, the SQRTPA defines permanent hypoparathyroidism based on the need for active vitamin D and/or calcium supplementation, without considering biochemical data on calcium and PTH levels; this differs from the definition used in the present study. Finally, the poor concordance between the SQRTPA and the present study may to some extent be explained by erroneous input or data missing from the SQRTPA.

The strength of this study is the large number of patients and fact that it is population-based. A strict definition of definitive permanent hypoparathyroidism was used, including data on both PTH and calcium values, as well as documented attempts to stop the treatment, making the diagnosis of permanent hypoparathyroidism highly reliable.

However, the study has several limitations. There may be bias and confounding factors because of the retrospective study design. A structured protocol was used to reduce the risk of misclassification bias. It is nevertheless possible that some information was missing from the charts. For example, although data on the number of parathyroids identified during surgery were available, data on incidentally removed parathyroids found in the specimens were not available. There was a large amount of missing information on postoperative laryngoscopy, making the data on RLN injury uncertain. Routines for postoperative laryngoscopy differed among the participating hospitals throughout the study interval, and not all patients would have undergone postoperative laryngoscopy, given that there were no concerns about voice quality after surgery. Furthermore, the PTH level was not checked routinely at the start of the study interval, and availability differed between the hospitals during the study. The methods used for PTH analysis also varied between hospitals and throughout the study. For this reason, PTH was categorized as being either below the lower limit of the reference range, or within or above the normal reference range.

The risk of permanent hypoparathyroidism after total thyroidectomy is high. Some patients are misdiagnosed and overtreated because attempts to stop the treatment are not made. The rate of low PTH level in the first 24 h after surgery might be a promising surrogate for the rate of permanent hypoparathyroidism in a cohort. Long-term follow-up data on surgical complications might be underestimated in quality registers.

## Supplementary Material

znad366_Supplementary_Data

## Data Availability

The data that support the findings of this study are available on request from the corresponding author. The data are not publicly available owing to restrictions, for example containing information that could compromise the privacy of research participants.
