# Supplementary material for: Validating the risk of hypoparathyroidism after total thyroidectomy in a population-based cohort: plea for improved follow-up
Source: Br J Surg. 2023 Nov 23;111(1):znad366. doi: 10.1093/bjs/znad366 (PMC10776524; doi:10.1093/bjs/znad366)
Supplement: znad366_Supplementary_Data [file znad366_supplementary_data.docx]

**Title: Validating the risk of hypoparathyroidism after total thyroidectomy in a population-based cohort: a plea for improved follow-up**

Authors: Matilda Annebäck MD PhD^1^, Carolina Osterman MD ^2^, Jesper Arlebrink MD ^3^, Simon Mellerstedt MD ^4^, Nicolas Papathanasakis MD ^5^, Göran Wallin MD PhD^5^, Ola Hessman MD PhD^6^, Maria Annerbo MD PhD^4^, Olov Norlén MD PhD^1^

^1^Department of Surgical Sciences, Uppsala University, Uppsala, Sweden

^2^Department of Surgery, Gävle Hospital, Gävle, Sweden

^3^Department of Surgery, Karlstad Central Hospital, Karlstad, Sweden

^4^Department of Surgery, Falu Hospital, Karlstad, Sweden

^5^Department of Surgery, Örebro University Hospital, Örebro Sweden

^6^Department of Surgery, Västerås Central Hospital, Västerås, Sweden

**Corresponding author.** Matilda Annebäck, MD, PhD, Department of Surgical Sciences, Uppsala, University Hospital SE-751 85 Uppsala, Sweden.

**Supplementary Materials - Index**

| **Supplementary Figures and Tables** |  |
| --- | --- |
| Figure S1. Map over Sweden and participating hospitals with their catchment area and number of patients included | *page 8* |

**Supplementary Figures and Tables**

**Figure S1.** Map over Sweden and participating hospitals with their catchment area and number of patients included

**
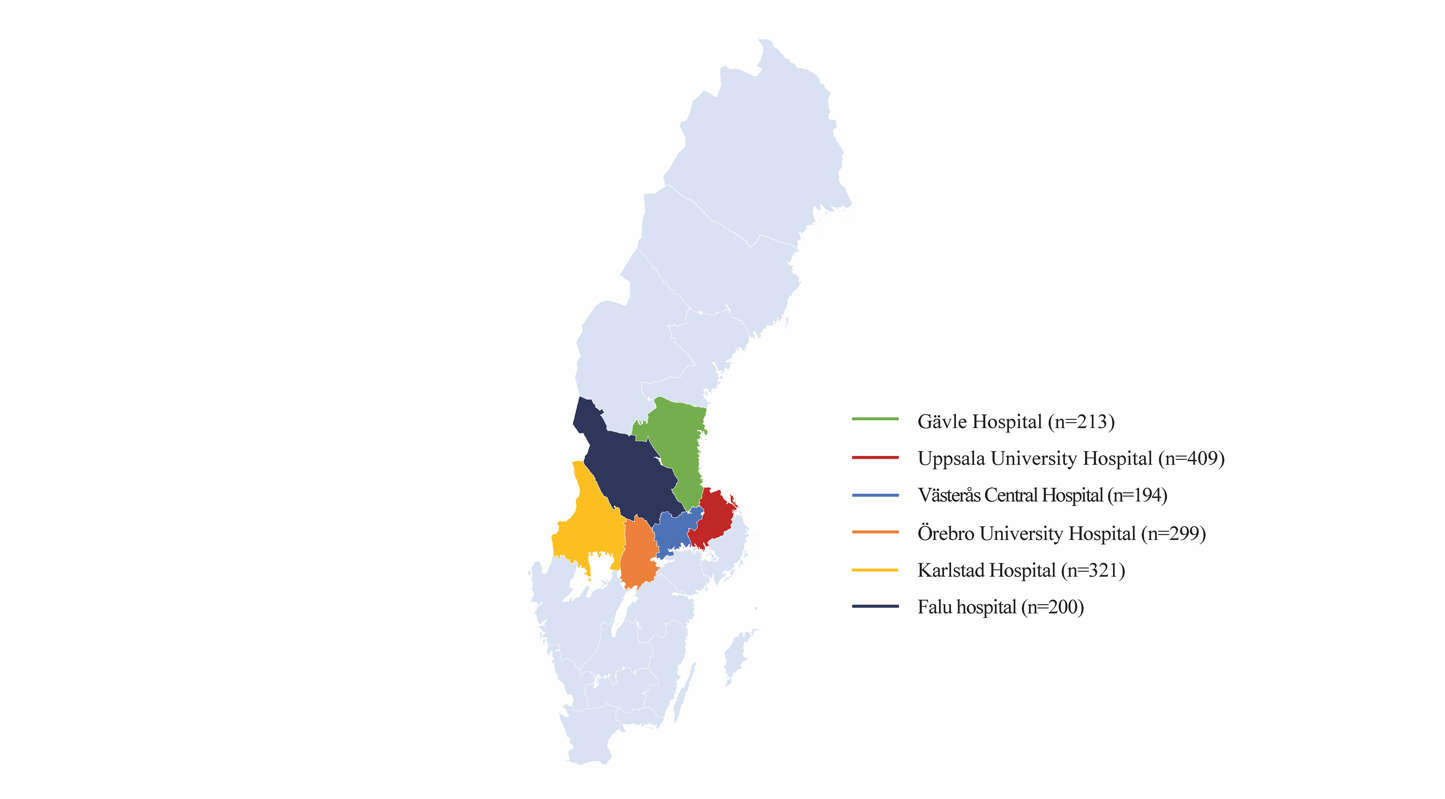
**
